# Effect of *Salmonella* pathogenicity island 1 and 2 (SPI-1 and SPI-2) deletion on intestinal colonization and systemic dissemination in chickens

**DOI:** 10.1007/s11259-023-10185-z

**Published:** 2023-07-25

**Authors:** Jwerlly Tatiana Pico-Rodríguez, Hugo Martínez-Jarquín, José de Jesús Gómez-Chávez, Mireya Juárez-Ramírez, Luary Carolina Martínez-Chavarría

**Affiliations:** Departamento de Patología, Facultad de Medicina Veterinaria y Zootecnia, Universidad Nacional Autónoma de México, Coyoacán, Ciudad de México, 04510 México

**Keywords:** *Salmonella*, Pathogenicity islands, SPI, Chicken, Histopathology, Immunohistochemistry

## Abstract

*Salmonella*’s virulence genes are located in two regions known as *Salmonella* pathogenicity islands 1 and 2 (SPI-1 and SPI-2). SPI-1 allows the bacteria to invade the intestine, while SPI-2 is important for intracellular survival and replication, although it is also necessary for intestinal disease. The aim of this study was to evaluate the effect of the deletion of SPI-1 or SPI-2 genes on the intestinal and systemic salmonellosis using the avian model. Groups of chickens were orally infected with 10^10^ Colony-Forming Units (CFU) of *S*. Typhimurium SL1344 WT strain, as well as mutants ∆SPI-1 or ∆SPI-2. At different times post-infection, 5 chickens from each group were euthanized and examined *postmortem*. Cecum and liver were taken from each chicken for determination of CFU’s, histopathological analysis and immunochemistry. Bacterial colonies were recovered from the liver and cecum samples infected with WT strain, while in the cultures from the organs infected with the mutant strains no colonies were recovered or were drastically affected in the ability to survive. In histopathological analysis, the WT strain produced lesions in liver and ceca, and it was detected by immunohistochemistry throughout the course of the infection. On the other hand, organs of chickens infected with ∆SPI-1 or ∆SPI-2 showed attenuated lesions and the immunohistochemistry revealed less bacteria compared to the WT strain. Taken together, our results show the importance of SPI-1 and SPI-2 genes for the complete intestinal and systemic disease in an in vivo avian model.

**Supplementary Information:**

The online version contains supplementary material available at 10.1007/s11259-023-10185-z.

## Introduction

The ability of *Salmonella* to invade and colonize various cell types within its host (including non-phagocytic intestinal epithelial cells, and phagocytic cells such as macrophages), and to cause disease, depends on its virulence genes. Most of these virulence genes are located within specific regions of the genome known as *Salmonella* Pathogenicity Islands (SPI) (Marcus et al. 2000; Dos Santos et al. 2019; Jajere 2019). To date, five SPIs have been described in *Salmonella* Typhimurium, with islands 1 and 2 (SPI-1 and SPI-2) being the most recognized and studied (Dos Santos et al. 2019). Both SPI-1 and SPI-2 contain genes encoding a Type Three Secretion System (TTSS), through which the bacterium translocates different effector proteins into the host cell (Hansen-Wester and Hensel 2001; Jennings et al. 2017; Lou et al. 2019).

The main function of SPI-1 effector proteins is to allow invasion into enterocytes, while SPI-2 effector proteins allow *Salmonella* to survive intracellularly, and, subsequently, to spread systemically to other organs, such as the liver and spleen (Jennings et al. 2017; Lou et al. 2019; Haraga et al. 2008).

Multiple animal models have been used to study *Salmonella* pathogenesis, such as susceptible mouse lineages, mice pre-treated with streptomycin, bovine ligated ileal loops, zebrafish, and the nematode *C. elegans*, among others (Santos et al. 2001; Barthel et al. 2003; Paulander et al. 2007; Costa et al. 2012; Howlader et al. 2016; Ehrhardt and Grassl 2022). The most widely used animal model is the mouse model; however, in mouse model of colitis the pre-treatment with streptomycin alters the microbiota.

Previous studies have used chickens to study the *S*. Typhimurium disease because depending on the age of the birds, intestinal disease (1-week-old chickens) or systemic infection (1-day-old chickens) can be detected (Beal et al. 2004; Withanage et al. 2004, 2005). Furthermore, other studies have examined the role of SPI-1 and SPI-2 in these animals; however, some of them have involved mutants affected only in one SSTT structural gene encoded on each island (Wigley et al. 2002; Dieye et al. 2009; Jones et al. 2007) and those which have used mutants lacking the whole SPI1 or SPI2 islands have used other serotypes, such as *S.* Enteritidis (Desin et al. 2009; Richlik et al. 2009; Wisner et al. 2010; Matulova et al. 2012; Eade et al. 2018) or *S.* Pullorum (Wigley et al. 2002). The aim of this study was to evaluate the course of a *S*. Typhimurium infection in chickens, when the bacteria lacks all the SPI-1 or SPI-2 genes.

## Materials and methods

### Bacterial strains and growth conditions

Bacterial strains used in this work were *S.* Typhimurium SL1344 (WT, Str^R^) and its derivative mutants ΔSPI-1 (SPI-1::Km, Km^R^) and ΔSPI-2 (SPI-2::Km, Km^R^). To do the infections, bacterial cultures were grown overnight at 37° in LB medium in an orbital shaking incubator (Incushaker mini; Benchmark) at 200 rpm. The next day these cultures were transferred to another culture and were grown for 5–6 h under the same conditions; subsequently the cultures were concentrated by centrifugation. Cultures were supplemented with streptomycin (100 μg/ml) or kanamycin (50 μg/ml).

### Experimental animals

One-day-old specific-pathogen-free chickens were purchased from ALPES (Puebla, Mexico). Birds were housed in separate cages at 30° C, which was reduced to 20° C when the chickens reached 1 week of age. Water and food were provided *ad libitum* following infection.

### Chicken infection experiments

Sixty 1-day-old chicks were divided into four equal groups and orally infected with 10^10^ colony-forming units (CFUs) of the WT strain, the ΔSPI-1 or ΔSPI-2 mutant strains. Another group was inoculated as control with PBS. At 24-, 48- and 72-hours post infection (hpi), five chickens from each group were euthanized for further *postmortem* analysis, as previously reported (Jones et al. 2007).

Sixty 1-week-old chickens were divided into four equal groups and they were infected as described above. At 1-, 3-, and 7-days post infection (dpi), five chickens from each group were euthanized for further *postmortem* analysis, as previously reported (Jones et al. 2007).

At *postmortem* analysis, liver and ceca were removed aseptically and processed for bacteriological, histopathological and immunohistochemical analysis.

### Bacterial counts

Chicken liver and cecum were collected and homogenized in sterile PBS. Serial dilutions of the homogenized organs were plated on LB agar and Brilliant Green agar, with the addition of 100 μg/ml streptomycin for the WT strain, and 100 μg/ml kanamycin for the mutant strains and incubated at 37° C for 24 h.

### Histopathological analysis

The livers and cecum harvested from experimental animals were fixed in 10% neutral buffered formalin for 24 h. After that, they were embedded in paraffin to be subsequently stained with hematoxylin and eosin (H&E) for the lesions analysis, and with Gram stain to detect bacteria. For cecum samples, apoptotic bodies, vacuolar degeneration and exocytosis, were analyzed and scored as follows: absent = 0, scarce = 1, moderate = 2, abundant = 3. The same values were used to score liver necrotic foci and inflammatory infiltrate. For the inflammatory infiltrate in the cecum, it was assessed in which layer the heterophiles were found (epithelium, lamina propria, basal membrane, submucosa, and organ muscle).

### Polymerase chain reaction (PCR)

DNA was isolated from bacterial cultures, as well as paraffin-embedded organs using the DNeasy Blood & Tissue commercial kit (Qiagen, Germany) according to the manufacturer’s instructions. The extracted DNA was used as a template for carrying out the PCR. To identify ΔSPI-1 mutant, a gene from SPI-2 (*ssrB*, 459 bp), was amplified. To identify ΔSPI-2 mutant, a gene from SPI-1 (*hilA,* 441 bp), was amplified. To identify the WT strain, we amplified any of those genes (*hilA* or *ssrB*).

PCR reactions were performed in a final volume of 25 μl containing: 12.5 μl of TopTaq Master Mix (1.25 units TopTaq DNA polymerase, 1× PCR buffer with 1.5 mM MgCl2, 200 μM of each dNTP), 1.5 μl of each primer at a concentration of 25 pmol and ~ 100 ng of the extracted DNA. The thermocycling profile consisted of an initial denaturing step at 94° C for 5 min, followed by 35 cycles of denaturation for 1 min at 94° C, annealing at 62° C for 1 min, and extension at 72° C for 1 min. At the end of the cycling, a final extension step was carried out at 72° C for 10 min. PCR products were separated by electrophoresis through a 2% agarose gel, visualized and documented on a UVP UVsolo touch (Analytik Jena, Germany).

### Immunohistochemistry (IHC)

Cecum and liver samples embedded in paraffin were dewaxed at 55° C for 30 min, rehydrated twice in xylol and ethanol at different concentrations (100%, 96%, 80% and 70%), and washed in distilled water. Endogenous peroxidase activity was inhibited by placing the slides in hydrogen peroxide and methanol (3ml/100ml) for 20 min at room temperature (RT). For antigen retrieval, slides were included in heat retrieval solution Diva Decloaker 20X (BioCare Medical), and placed in a pressure cooker for 3 min, tempered for 5 min, and washed with PBS-TWEEN 0.1%. Following the blocking, the slides were incubated with Background Sniper (BioCare Medical) for 15 min at RT and washed with PBS-TWEEN 0.1%. Then, a primary polyclonal anti-*Salmonella* Typhimurium antibody (BIOSS) diluted 1:300 was applied to the slides, incubated for 2 h at RT, and washed in PBS-TWEEN 0.1%. Subsequently, the slides were exposed to HRP-Polymer (Biocare Medical, USA) for 30 min, washed with PBS-TWEEN 0.1% and, finally, diaminobenzidine (Betazoid DAB Chromogen- Biocare Medical, USA) was applied for 1 min to detect the primary antibody.

### Statistical analysis

Bacterial load was compared using analysis of variance with Tukey’s multiple-comparisons posttest. Pathological scores were compared using the Generalized Linear Model (GzLM), introducing lesions as a response variable, as well as time and strain as independent variables, with significant differences (P ≤ 0.05). The Bonferroni method was used to determine the difference between pairs. Statistical analysis was conducted using IBM SPSS Statistic 28 software.

## Results

### Deletion of SPI-1 or SPI-2 genes affects the ability of ***Salmonella*** to be recovered from cecum and liver in 1-day-old and 1-week-old chickens

**Fig. 1:**
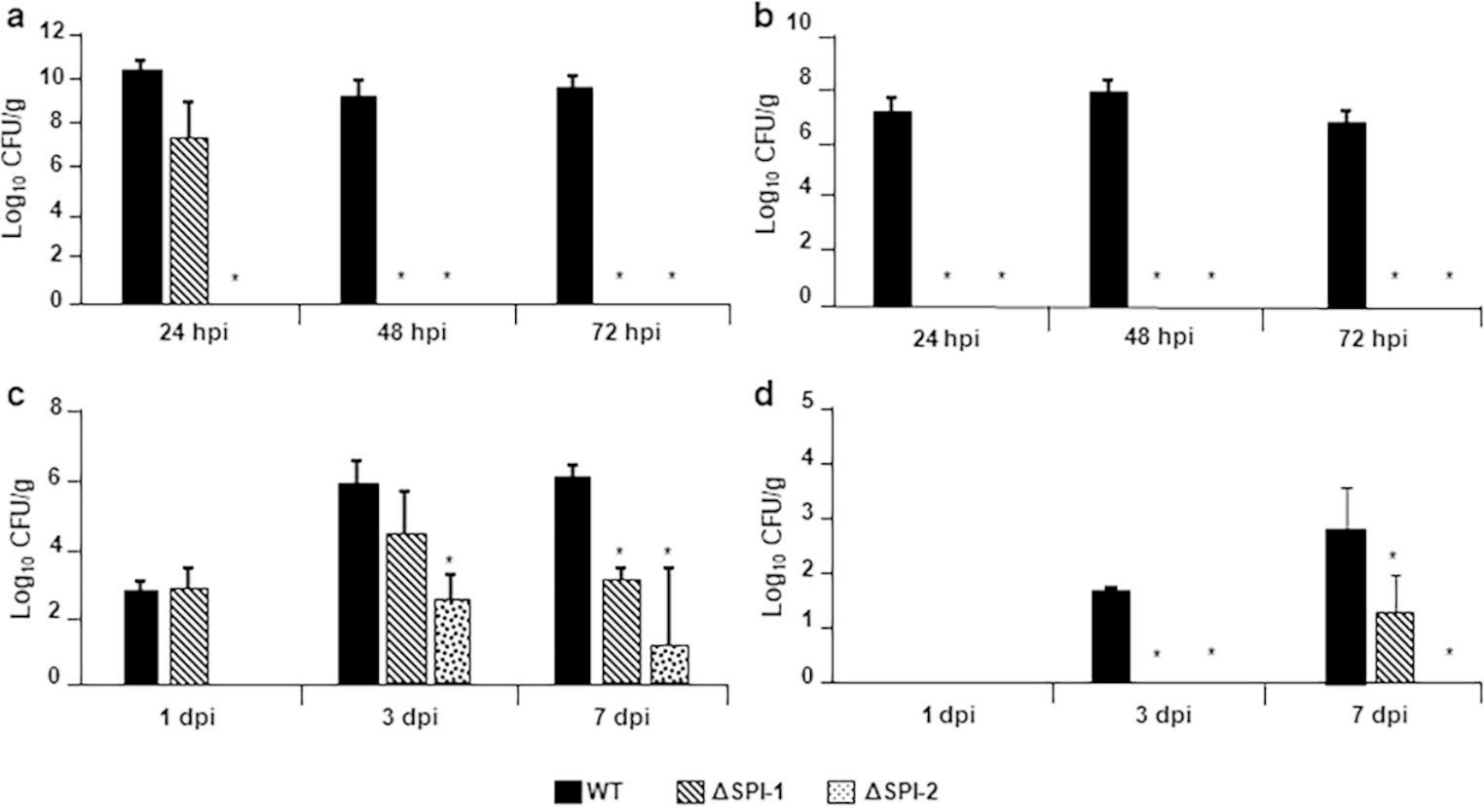
CFU bacterial counts per gram. Bacterial loads from cecal content (**a**) and liver (**b**) of 1-day-old chickens at 24-, 48- and 72 h post infection. Bacterial loads from cecal content (**c**) and liver (**d**) of 1-week-old chickens at 1-, 3- and 7 days post infection. Error bars indicate the standard deviation. *Significant differences between mutants and WT strain (P ≤ 0.05)

In 1-day-old chickens, the WT strain was recovered from ceca contents in similar levels (10^9^-10^10^ CFU/g) at 24-, 48- and 72-h post-infection; while the ΔSPI-1 strain was recovered in lower amounts at 24 hpi (10^7^ CFU/g), and was not recovered at 48 and 72 h. The ΔSPI-2 strain was not recovered at any time of infection (Fig. 1a). In the liver, WT strain was also recovered in similar levels at 24-, 48-, and 72-h post-infection (10^7^-10^8^ CFU/g), while these levels were lower than those observed in the cecum (Fig. 1a and b). In contrast, no mutant strain was recovered from this organ at any sampling time (Fig. 1b).

In ceca from 1-week-old chickens, WT strain were recovered in low amounts (10^3^ CFU/g) at 1-day post-infection (dpi) and these numbers increased along the infection, being 10^6^ at 3 and 7 dpi (Fig. 1c). These amounts were considerably lower than those observed with the WT strain in ceca of 1-day-old chickens (10^10^) (Fig. 1a). ΔSPI-1 was recovered in similar amounts to the WT during the first 3 dpi but its growth was affected at 7 dpi in comparison with the WT strain (Fig. 1c). ΔSPI-2 was more affected than ΔSPI-1 as it was only recovered at 3 and 7 dpi and in lower amounts than the WT (Fig. 1c).

In the liver samples, WT strain recovery was increased along the infection (Fig. 1d), but the amounts were significatively lower to those observed in livers from 1-day-old chicken (10^8^ vs. 10^3^) (Fig. 1b and d). ΔSPI-1 was only recovered at 7 dpi but the amounts were lower than those observed in WT strain, while ΔSPI-2 was not recovered at any time (Fig. 1d).

Taken together, these results show that deletion of SPI-1 or SPI-2 genes affected the ability of *S.* Typhimurium to colonize the cecum and disseminate to the liver, being this effect more drastic in 1-day-old chickens.

### WT strain progressively showed significant injury in cecum and liver of 1-day-old and 1-week-old chickens

**Fig. 2:**
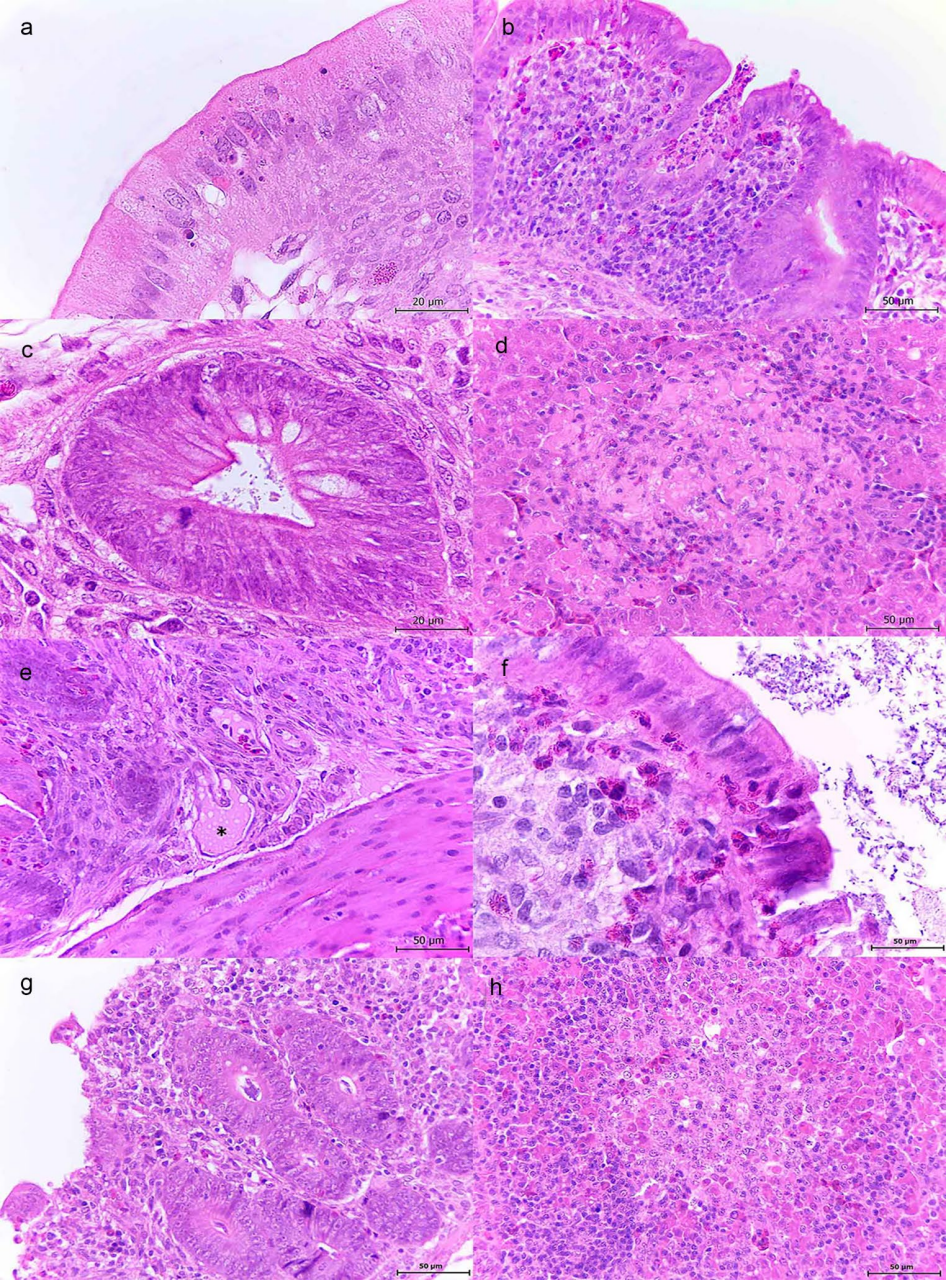
Histopathological features observed in cecum and liver from 1-day-old and 1-week-old chickens infected with wild-type *S*. Typhimurium. 1-day-old infected chickens (a-d). **a**: Cecum. Light eosinophilic cytoplasm (vacuolar degeneration) and nuclear debris (apoptotic bodies) are observed in some enterocytes. H&E 100x. **b**: Cecum. Thickened lamina propria due to lymphocytic and heterophilic infiltrate. Abundant degenerated and necrotic heterophils are stuck in the crypt and projected to the intestinal lumen. H&E 40x. **c**: Cecum. Bacilli are observed in the crypts as well as adhered to the enterocytes. H&E 100x. **d**: Liver. Areas of hepatic necrosis surrounded by heterophils and lymphocytes. H&E 40x. 1-week-old infected chickens (e-h). **e**: Cecum. Lamina propria showing dilated lymphatic vessels (asterisk). H&E 40x. **f**: Cecum. Aggregates of heterophils are observed among the enterocytes and in the lamina propria. Presence of bacilli in the intestinal lumen and attached to the enterocytes. H&E 100x. **g**: Cecum. Superficial epithelium erosion and aggregates of lymphocytes and heterophils in the lamina propria. H&E 40x. **h**: Liver. Heterophils and lymphocytes surrounding areas of hepatic necrosis. H&E 40x

1-day-old chickens. Cecum of the WT infected chickens showed slight morphological changes at 24 hpi, such as apoptotic bodies and vacuolar degeneration (Fig. 2a). At 48 hpi, these lesions were markedly increased (Table 1); the lamina propria was expanded by lymphocytes and heterophils; volcano-like lesions consisting of multiple degenerated and necrotic heterophils were observed arising from the crypts (Fig. 2b) and bacteria were seen in the lumen, crypts (Fig. 2c), as well as adhered to the epithelium. At 72 hpi, the lesions were more severe; the lymphoplasmacytic and histiocytic infiltrate was distributed throughout the lamina propria, and transmurally in some chicks (Table 1). At this point bacteria surrounded necrotic foci, and *Salmonella*-containing vacuoles were seen inside the enterocytes; Gram stain demonstrated the presence of Gram negative bacilli (Fig. S1) which were confirmed as *Salmonella* by PCR (data not shown).

**Table 1:** Histopathology scores from caecum and liver from 1-day-old chickens

Samples	Findings	24 hpi			48 hpi			72 hpi		
		WT	ΔSPI-1	ΔSPI-2	WT	ΔSPI-1	ΔSPI-2	WT	ΔSPI-1	ΔSPI-2
**Caecum**	**Apoptotic bodies**	2.25 ± 0.96	0^a^	0^a^	1.75 ± 0.96	0^a^	0^a^	3 ± 0	0^a^	0^a^
	**Vacuolar degeneration**	0.75 ± 1.50	0^a^	0^a^	3 ± 0	0^a^	0^a^	3 ± 0	0^a^	0^a^
	**Exocytosis**	0.75 ± 0.50	0^a^	0^a^	1.75 ± 0.96	0^a^	0^a^	3 ± 0	0^a^	0^a^
	**Inflammatory infiltrate**	EP/LP/BM	---	---	EP/LP/BM/SM	---	---	EP/LP/BM/SM/OM	---	---
**Liver**	**Necrosis**	0	0	0	1.25 ± 0.50	0^a^	0^a^	2 ± 1	0^a^	0^a^
	**Inflammatory infiltrate**	0	0	0	0.75 ± 0.50	0^a^	0^a^	1.7 ± 0.58	0^a^	0^a^

EP = Epithelium LP = Lamina propria BM = Basal membrane SM = Submucosa OM = Organ muscle ^a^ Superscript letter indicates significant differences with the WT strain (P < 0.05)

In the liver of chickens infected with WT strain, necrotic foci, dissociated hepatic cords and inflammatory infiltrate were observed starting at 48 hpi and increased at 72 hpi (Table 1). Necrotic debris were surrounded by lymphocytes, heterophils and macrophages (Fig. 2d).

1-week-old chickens. Chickens inoculated with WT strain displayed apoptotic bodies in enterocytes, heterophils in all layers of the intestine (Table 2), and variable amounts of intraluminal and intracryptal bacteria were seen at 1 dpi. At 3 and 7 dpi, apoptotic bodies and some sections with dilated lymphatic vessels were seen (Fig. 2e). In the lamina propria and among the enterocytes, aggregates of heterophils were observed; bacilli were seen in the lumen, adhered to the epithelium, and associated to areas of erosion and ulcers (Fig. 2f). Lamina propria was thickened by lymphocytic and heterophilic infiltrate (Fig. 2g). In some chicks, dilated blood vessels with septic emboli could be observed.

**Table 2:** Histopathology scores from caecum and liver from 1-week-old chickens

Samples	Findings	1 dpi			3 dpi			7 dpi		
		WT	ΔSPI-1	ΔSPI-2	WT	ΔSPI-1	ΔSPI-2	WT	ΔSPI-1	ΔSPI-2
**Caecum**	**Apoptotic bodies**	3 ± 0	1.5 ± 0.5^a^	0^a^	2.25 ± 1.50	2.25 ± 0.50	2 ± 0.82	3 ± 0	2 ± 0.82	3 ± 0
	**Vacuolar degeneration**	2.25 ± 1.50	0^a^	0^a^	1.5 ± 1.73	1.75 ± 0.96	1 ± 0	3 ± 0	1.25 ± 1.26	2 ± 0.82
	**Exocytosis**	1.75 ± 1.26	0^a^	0^a^	1.75 ± 0.50	0^a^	0^a^	1.25 ± 0.96	0	0.5 ± 1
	**Inflammatory infiltrate**	EP/LP/BM/SM	---	---	EP/LP/BM/SM/OM	---	---	EP/LP/BM/SM/OM	---	---
**Liver**	**Necrosis**	2.25 ± 0.50	0^a^	0^a^	3 ± 0	0^a^	0^a^	3 ± 0	0^a^	0^a^
	**Inflammatory infiltrate**	1.75 ± 0.96	0^a^	0^a^	2.25 ± 0.50	0^a^	0^a^	2.5 ± 0.58	0^a^	0^a^

EP = Epithelium LP = Lamina propria BM = Basal membrane SM = Submucosa OM = Organ muscle ^a^ Superscript letter indicates significant differences with the WT strain (P < 0.05)

In the liver, extensive areas of necrosis with lymphocytes, degenerated heterophils, and some areas of mild hemorrhage were observed dissociating the hepatic cords of this group of birds. These lesions became worse as the time of infection progressed (Table 2; Fig. 2h).

### ∆SPI-1 and ∆SPI-2 do not produce cecal and hepatic lesions in 1-day-old chickens, while in 1-week-old chickens they induced scare or moderate lesions at 3 and 7 days post infection

**Fig. 3:**
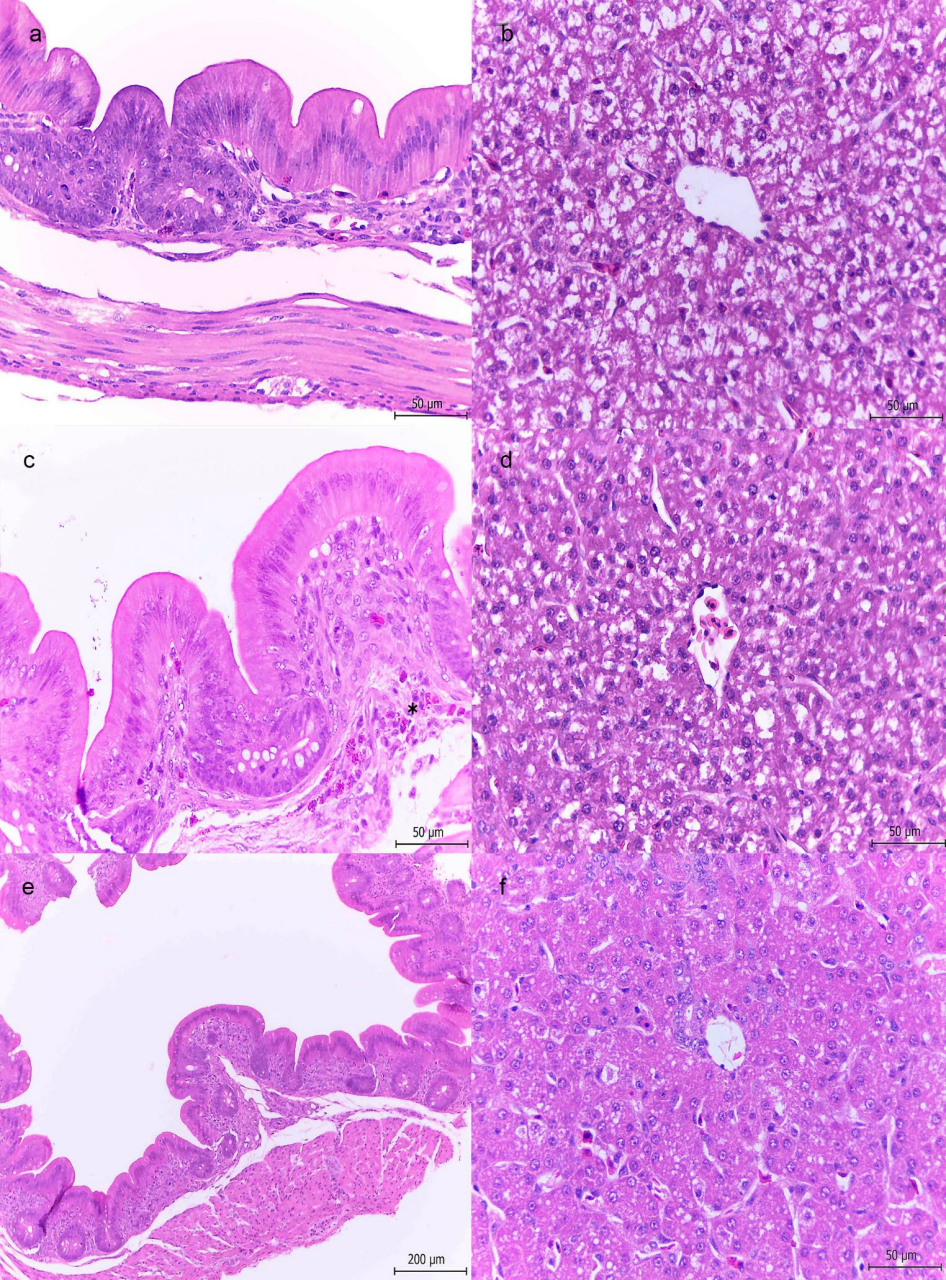
Histopathological features observed in cecum and liver from 1-day-old chickens infected with ΔSPI-1 or ΔSPI-2 mutant strains. **a**: Cecum, ΔSPI-1. Intestinal mucosa without pathological changes. H&E 40x. **b**: Liver, ΔSPI-1. Hepatocytes showing multiple cytoplasmic pale vacuoles (vacuolar change). H&E 40x. **c**: Cecum, ΔSPI-2. Heterophils aggregates are shown in the lamina propria (asterisk). H&E 40x. **d**: Liver, ΔSPI-2. Vacuolar change in the hepatocytes. H&E 40x. **e**: Cecum, control group. H&E 10x. **f**: Liver, control group. H&E 40x

1-day-old chickens. Chickens inoculated with ∆SPI-1 or ∆SPI-2 showed no lesions in ceca or liver at any time along the infection (Table 1; Fig. 3a-d).

**Fig. 4:**
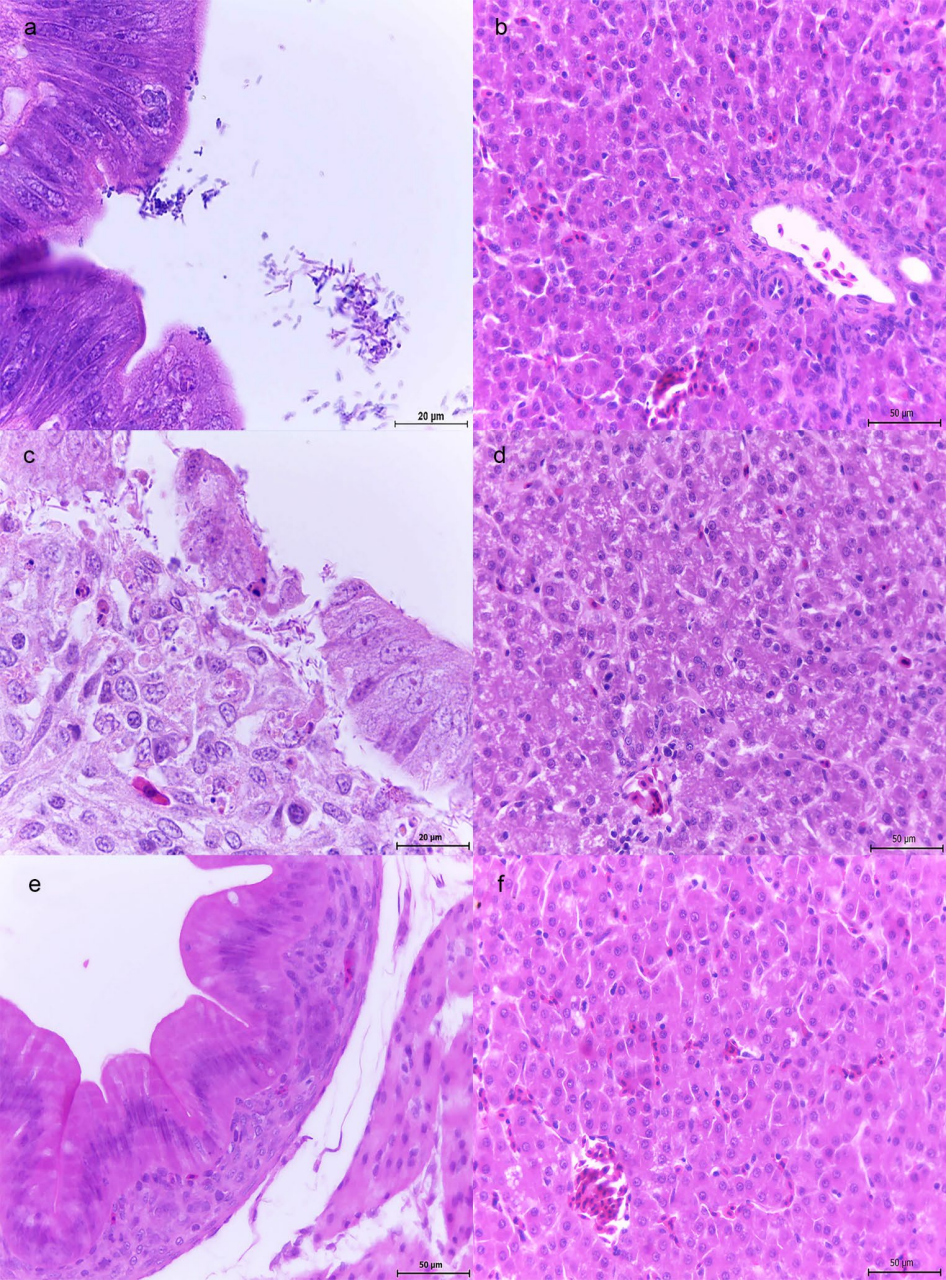
Histopathological features observed in cecum and liver from 1-week-old chickens infected with ΔSPI-1 or ΔSPI-2 mutant strains. **a**: Cecum, ΔSPI-1. Bacilli aggregates attached to the enterocytes and in the intestinal lumen. H&E 100x. **b**: Liver, ΔSPI-1. No pathological changes. H&E 40x. **c**: Cecum, ΔSPI-2. Bacteria in the lamina propria and attached to the enterocytes. Slight vacuolar degeneration in the enterocytes; apoptotic bodies in the epithelium and lamina propria. H&E 100x. **d**: Liver, ΔSPI-2. No pathological changes. H&E 40x. **e**: Cecum, control group. H&E 40x. **f**: Liver, control group. H&E 40x

1-week-old chickens. At 1 dpi chickens inoculated with either ∆SPI-1 or ∆SPI-2 strain showed scant or no lesions in cecum (Table 2). At 3 and 7 dpi, ∆SPI-1 was only observed in the intestinal lumen or adhered to the epithelium but not inside the enterocytes (Fig. 4a), whereas ∆SPI-2 was also observed intralesionally in areas of mild ulceration (Fig. 4c). Enterocytes showed slight vacuolar degeneration; some apoptotic bodies were seen in the epithelium and lamina propria (Fig. 4c). No liver lesions were observed in any chicken infected with either mutant at any time along the infection (Table 2; Fig. 4b and d). No bacteria or lesions were found in the control groups at any time as observed in Figs. 3e-f and 4e-f.

### WT and both mutant strains are differently immunolocated in cecum or liver along the infection in 1-day-old and 1-week-old chickens

**Fig. 5:**
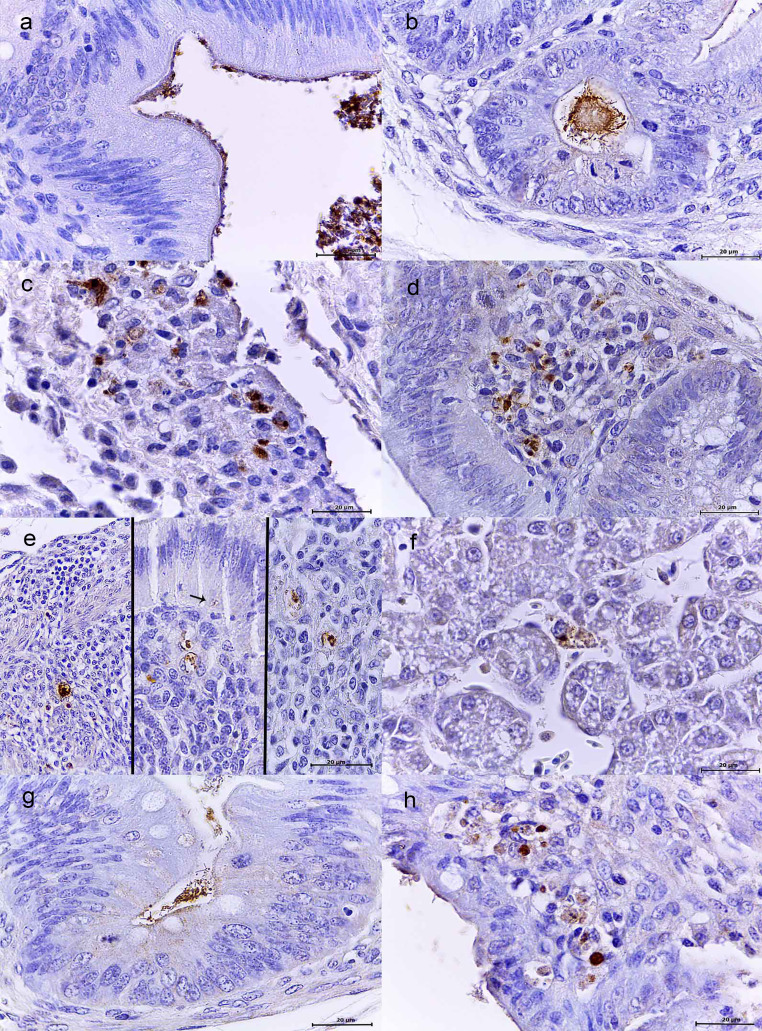
Immunostaining with polyclonal anti-*Salmonella* Typhimurium antibody. (a-e) WT, cecum. *S*. Typhimurium was attached to the epithelium and the intestinal lumen (**a**), as well as inside the crypts (**b**). As infection progressed, it was detected inside macrophages and among necrotic debris in the lamina propria (**c,d**). **e**: Bacteria detected inside intracytoplasmic vacuoles in enterocytes (arrow) and macrophages. **f**: WT, liver. *S*. Typhimurium inside hepatocytes. **g**: ΔSPI-1, cecum. Bacteria attached to the epithelium and inside crypts. **h**: ΔSPI-2, cecum. Bacteria were detected in the lamina propria and inside enterocytes. Indirect immunochemistry with the EnvisionFLEX-HRP detection system, contrasted with Harris’ hematoxylin. 100x

In order to specifically determine the tissue location of *Salmonella* Typhimurium throughout the infections, we detected the bacteria by immunohistochemistry in the same samples where the bacteria were detected by histopathology in cecum and liver. In both the 1-day-old and 1-week-old chickens, WT strain was detected throughout the whole infection. At 24 hpi, it was detected in the intestinal lumen and adhered to the enterocytes (Fig. 5a). As the infection progressed it was possible to detect it inside crypts (Fig. 5b) and enterocytes. Later, it was also multifocally distributed throughout the lamina propria, and macrophages (Fig. 5c and d). In these cells as well as in enterocytes, it was possible to detect the bacteria inside intracytoplasmic vacuoles (Fig. 5e).

In liver samples from 1-day-old and 1-week old chickens, WT was observed inside hepatocytes and associated with degenerated and necrotic hepatocytes areas (Fig. 5f), in agreement with the CFUs counts and histopathology analysis.

The ΔSPI-1 mutant was only detected attached to the epithelium and inside the crypts in 1-day-old and 1-week-old chickens (Fig. 5g). In 1-day-old chickens this mutant did not cause any damage, and in 1-week-old chickens this strain was observed in areas of scarce to moderate lesions which correlates with the histopathology values presented in Table 2.

In samples from 1-day-old chickens, ΔSPI-2 strain was not detected at any time, in agreement with the CFUs counts and histopathology results (Fig. 1a; Table 1). In 1-week-old chickens this mutant was detected in the lumen, crypts, ulcerative lesions, enterocytes as well as in the lamina propria (Fig. 5h), which correlates with histopathology (Fig. 4c).

In the livers of 1-day-old as well as 1-week-old chickens, neither ΔSPI-1 nor ΔSPI-2 mutants were detected along the infections, which is consistent with the absence of lesions at any time (Tables 1 and 2).

These results demonstrate that WT strain can be located in cecum and liver lesions along the infections. In contrast, ΔSPI-1 was not able to invade cecum and reach the liver whereas ΔSPI-2 could be located inside cells, but it induced less severe lesions than the WT and was not able to reach the liver.

## Discussion

The avian model allows the study of the different clinical presentations of *Salmonella* Typhimurium; here we used 1-day-old and 1-week-old chickens to evaluate the effect of the two most important pathogenicity islands for *Salmonella*, SPI-1 and SPI-2, using two mutants which have lost all the genes of each island.

In 1-day-old chickens, WT strain was able to grow and replicate in constant amounts both in intestine and liver, although it was evident that bacterial counts recovered from the liver were lower than those recovered from the cecum, in agreement with previous reports (Jones et al. 2007). In 1-week-old chickens bacteria grew gradually along the infection and in lower amounts than those recovered from 1-day-old chickens. It has been reported that intestinal tract in newly hatched chicks is lowly populated, and the development of the gut microbial community may accelerate or potentiate the maturation of the gut immune system and increase its resistance to infection with different pathogens (Crhanova et al. 2011). Moreover, in neonatal life, both in mammals and in many avian species, the immune system takes longer to develop, so that they have poor immune responses at birth (Smith et al. 2022).

Therefore, our results show an age-dependent susceptibility to infection, as 1-day-old chickens allowed unrestricted *Salmonella* multiplication due to their immature immunological and microbial condition.

Our CFUs analysis show that the ΔSPI-1 and ΔSPI-2 mutants were drastically affected to be recovered from cecum and liver, in comparison to the wild-type strain in 1-day-old chickens; whereas in 1-week-old chickens although both mutants were affected in comparison with the WT, the effect of the absence of SPI-2 was more drastic. Jones et al. (2007) analyzed colonization in cecal contents and livers of 1-day-old and 1-week-old chickens infected with a wild-type strain of *S.* Typhimurium F98 (WT), or with mutants of SPI1 (*spaS*) or SPI2 (*ssaU*). In 1-day-old chickens they recovered similar counts of the WT and both mutants in the cecal contents, while in the liver they only found differences between mutants and WT at 48 and 72 h. Differences observed in the mutants cannot be attributed to the fact that both studies used different WT parental strains (F98 and SL1344) to construct the mutants, as WT were recovered in similar amounts in both studies. However, their mutants only lack the function of SpaS or SsaU, which are part of the TTSS of SPI-1 or SPI-2, respectively, whereas our mutants lacked the whole SPI-1 or SPI-2, avoiding any redundant effect that could compensate the lack of only one gen.

It has been reported that *Salmonella* can be translocated through M cells in a SPI-1-independent way (Martínez-Argudo and Jepson 2008). This could explain why we recovered ΔSPI-1 from the cecum only at 24 hpi. However, although this mutant could overcome the lack of SPI-1, once inside the cell, absence of SPI-1 effectors like SipB, which is responsible to induce macrophages apoptosis, render it susceptible to be destroyed and unable to grow and disseminate.

Dieye et al. (2009) used a coinfection approach to evaluate the effect of mutants lacking the whole SPI-1 and SPI-2 islands. They found that lack of SPI-2 does not affect the cecal colonization. Coinfections provide a sensitive assay, as they decrease the animal-to-animal variation; however, it has been speculated that WT bacteria could complement the defects of some mutants in *trans*, especially mutants in secreted virulence factors (Chiang et al. 1999). This could explain why they recovered both the WT and the ∆SPI-2 when they used a WT/∆SPI-2 coinfection.

Our results show the role of *S*. Typhimurium SPI-1 and SPI-2 genes to produce lesions in chickens. In 1-day-old chickens, both ΔSPI-1 and ΔSPI-2 mutants were drastically affected in their capacity to cause injury both in cecum and liver, as expected according to our UFCs analysis as we did not recover any mutant. On the other hand, in 1-week-old chickens, ΔSPI-1 and ΔSPI-2 mutants could not cause cecal lesions as severe as the wild type, despite both mutants were able to colonize the cecum. Our results show that regardless of the age of the chickens, the absence of SPI-1 or SPI-2 genes renders *S.* Typhimurium unable to disseminate and reach the liver.

Coburn et al. evaluated the role of SPI-1 and SPI-2 using streptomycin pretreated mice, which were infected with a WT strain (SL1344) or mutants affected in a gene of the TTSS of SPI-1 (*invA*) or SPI-2 (*ssaR*) (Coburn et al. 2005). They found that SPI-1 mutant was attenuated but SPI-2 was essential for complete virulence in murine infectious enterocolitis. Coombes et al. (2005) also evaluated these mutants in both the pretreated mice and bovine intestinal loops. In both models they found that SPI-1 mutant produced intestinal injuries similar to WT 5 dpi, whereas the SPI-2 mutant was dramatically attenuated. Their results show that although SPI-1 is important for the induction of enteritis early in infection, SPI-2 has a more important role in the pathogenesis of enteritis because it is required regardless of the presence of SPI-1. In agreement with those studies, our results provide evidence that SPI-2 is important for a complete intestinal disease and in addition they also show the importance of this island to produce lesions in chickens.

In contrast to Coombes et al. (2005) we found that SPI-1 mutant was attenuated at 7 dpi. As they used mutant strains affected only in one gene of the TTSS, there is still the possibility of any compensatory effect of other virulence factors contained in the SPIs.

Differences between our studies can be also attributed to the animal models. Pretreatment with streptomycin alters the microbiota and makes mice more susceptible to infection; whereas direct inoculation in the calves’ intestinal loops prevents the bacterial transit through upper sites in the bovine and this could over represent the amount of *Salmonella* that reaches the intestines in a natural infection. Both cases could mask the role of SPI genes in a natural infection.

Here we used IHC to follow for the first time the infection of *S*. Typhimurium in both 1-day-old and 1-week-old chicks. As the infection progressed, *S*. Typhimurium WT was located first in the cecal contents, attached to the epithelium, and then within enterocytes or macrophages, and finally within hepatocytes; this technique let us correlate the precise bacterial location with the bacterial amount recovered from organs and the development of lesions as the infection progressed. Similarly, Bellido-Carreras et al. (2019), used IHC to follow a *S*. Typhimurium infection but they used 4-week-old pigs and analyzed the bacterial location in the intestine, mesenteric lymph nodes and palatine tonsils for 1 month post infection. As expected, and in agreement with our results, as the infection progressed, they detected *S*. Typhimurium in deeper intestinal layers, and this was related to the amount of bacteria recovered from these organs. Interestingly, from 6 dpi they found progressive restoration of the epithelium, reduction of the inflammatory infiltrate and elimination of bacteria from the mucosa; in contrast, at this time post infection we found that the number of bacteria and pathological score were similar or increased as those detected earlier during the infection. Because we do not use the same animal model, these dissimilarities could be attributed to differences in the immune responses and innate defense mechanisms between the chickens and pigs. Being mammals, microbial colonization in pigs occurs at earlier life periods, from the gestation, birth canal during parturition, lactation, and close contact with the mother before weaning (Duarte and Kim 2022). These factors potentially have a critical impact also on the development of the immune system and this would render the pig more suitable to contend with the infection. As we did not extend the infection beyond 7 days, it would be interesting to analyze if signs of recovery can be observed also in chickens but at later stages of infection, due to differences inherent to the species.

We also used IHC to track both ΔSPI-1 and ΔSPI-2 mutants. Along the whole infections, ΔSPI-1 was impaired in its ability to invade, generate lesions, and therefore spread, which confirms the role of SPI-1 effectors for the invasion and induction of inflammation. ΔSPI-2 was not found at any time in 1-day old chickens, but it was found inside the cells in 1-week old chickens although it was unable to spread. Then, by having SPI-1, the ΔSPI-2 strain was still able to produce effector proteins involved in the invasion and initial inflammatory process in 1-week old chickens, but interestingly in 1-day old chickens the presence of SPI-1 was not enough for invasion which suggests that in these chickens *Salmonella* would require SPI-2 genes to induce the invasion along with the SPI-1 genes; it could be interesting to evaluate if this finding was driven by differences in the immune system and microbiota in 1-day-old chickens.

Our results demonstrate the importance of both SPI-1 and SPI-2 genes to produce a complete intestinal disease that subsequently leads to systemic dissemination, and they also show for the first time the exact location of *S.* Typhimurium through the avian infection. Taken together, they contribute to clarify the pathogenesis of the disease and confirm the suitability of the avian model for the study of salmonellosis.

### Electronic Supplementary Material

Below is the link to the electronic supplementary material.

**Supplementary Material 1: Fig. S1** Bacteria detection in cecal samples. Gram negative bacteria located inside the crypts (a) and in the injured cecal mucosa (b). Gram stain. 100x
