# Supplementary figures and images for: Effect of Salmonella pathogenicity island 1 and 2 (SPI-1 and SPI-2) deletion on intestinal colonization and systemic dissemination in chickens
Source: Vet Res Commun. 2023 Jul 25;48(1):49–60. doi: 10.1007/s11259-023-10185-z (PMC10811122; doi:10.1007/s11259-023-10185-z)

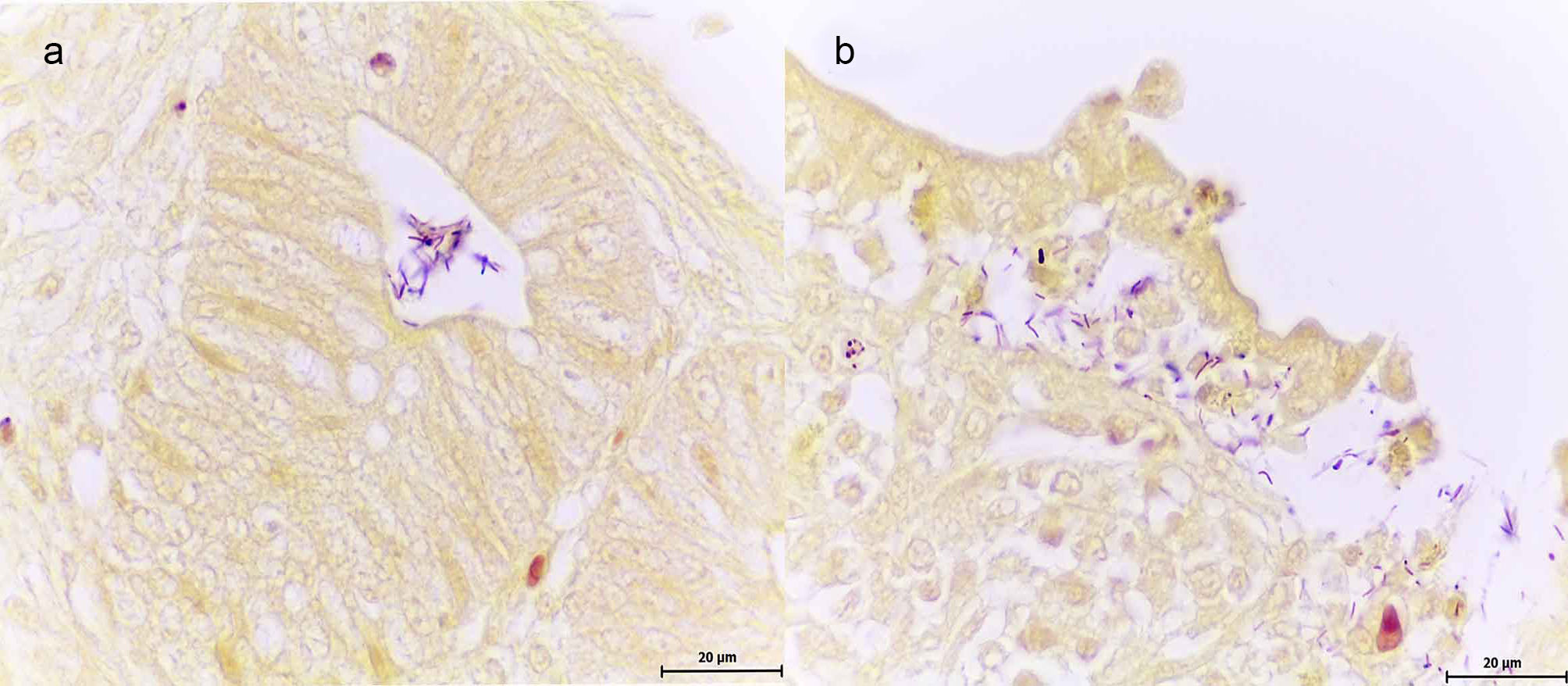

Supplement: Supplementary file 1 — Supplementary Material 1: Fig. S1 Bacteria detection in cecal samples. Gram negative bacteria located inside the crypts (a) and in the injured cecal mucosa (b). Gram stain. 100x [file 11259_2023_10185_MOESM1_ESM.png]
